# The Educational Community in the Face of COVID-19. Discursive Analysis on Vulnerability and Education

**DOI:** 10.3390/ijerph18136716

**Published:** 2021-06-22

**Authors:** Fernando López-Noguero, José Alberto Gallardo-López, Irene García-Lázaro

**Affiliations:** 1 Department of Education and Social Psychology, Pablo de Olavide University, 41013 Seville, Spain; flopnog@upo.es; 2 Department of Research and Diagnostic Methods in Education, University of Seville, 41013 Seville, Spain; igarcia9@us.es

**Keywords:** directorate of schools, COVID-19, educational challenges, social vulnerability, technology, digital divide

## Abstract

This article presents the results of research the objective of which was to know the opinion of directors of different educational centers about the management carried out in their centers and the possible difficulties encountered during the suspension of face-to-face classes caused by the first wave of the COVID-19 pandemic. The research method is qualitative, exploratory, descriptive, and inductive. The study sample is made up of 43 managers of educational centers, who were selected by applying an intentional sampling based on criteria of age, experience, ownership of the center, teachings that are taught, and the socioeconomic context where it is located. A questionnaire was used to collect the information, previously designed and validated through the judgment of experts, to inquire about the organization and management from the management team, the development of the academic task with the students, and the relationship with the families during the course period of cessation of classroom activities. To make an adequate approximation to the reality studied, an exhaustive content analysis of the speeches issued by the participants was carried out. Among the main conclusions of the research are the difficulties they have experienced during the closure of schools in relation to the lack of strategic planning to reconvert face-to-face education to the online format, scarcity of technological resources in centers and homes, training deficit in digital skills, increasing the digital divide, attention to students with special educational needs or communication problems with students and their families.

## 1. Introduction

The pandemic caused by the appearance of the SARS-CoV-2 (COVID-19) outbreak has caused a devastating impact on the health of citizens [1,2], but it is also having a significant impact on all countries of the world at the social level, economic, and cultural.

In an attempt to reduce its effects and prevent its rapid spread, most of the world’s governments have carried out a multitude of drastic and urgent measures to stop the spread of this highly contagious disease, imposing, among others, physical distancing, or the closure of schools and other teaching centers, moving, from one day to the next, to an online methodology in the education sector.

### 1.1. Impact on the Educational Community of the Suspension of Face-to-Face Classes

COVID-19 has forced educational institutions to re-organize in record time the teaching methodologies and evaluation systems originally developed to be carried out in person [3], having a great pedagogical, social, and psychological educational impact on teachers [4], as well as on students, families, and the educational community in general.

Thus, according to UNESCO [5], more than 1500 million students around the world were unable to continue their studies in person due to COVID-19. This figure means that 80% of the world’s children of school age have suffered and are currently suffering from the educational and social consequences derived from this pandemic, and they are a matter of concern for international organizations, national governments, schools, and the educational community in general.

When we speak of the educational community, we refer to the set of people who influence and are affected by a certain educational context (students, teachers, managers, and families, mainly); and that the educational process cannot be interpreted exclusively from the perspective of the teacher, but there are other social actors directly involved in this process [6,7,8].

The immediate closure of educational institutions [9,10,11] made educational agents have to adapt quickly to the situation, urgently applying a distance education model [12,13] for which the system was not necessarily ready. This scenario caused students to have to acquire new learning roles through information technology, this being the only means of interaction between teachers and students [14].

In Spain, the situation of a state of alarm was decreed on March 14 [15], establishing a school closure, which caused teachers, families, and students to face a completely unprecedented situation, never seen before [16]. To make this undertaking possible, teachers have had to rely on very diverse tools and digital resources, applying new approaches in the direction, management, communication, and teaching–learning process [17,18], where dialog between all parties involved in it becomes a transcendental element [19].

### 1.2. COVID-19 and the Challenge of ICT in Educational Contexts

The transition from face-to-face teaching to another alternative distance teaching model has not been an easy task, with very diverse difficulties, such as little training for its use, scarce technological resources, or, among other variables, restricted access by certain students to computer infrastructures [20,21,22,23]. It is evident that this situation has generated serious inequalities that affect the fundamental principle of equality in access and educational development.

A particularly negative aspect during this emergency situation caused by the COVID-19 pandemic has been the lack of interaction with students [24]. The fact is that the distance education model is a great challenge, and therefore, for its optimal development, it is necessary that there be immediate feedback supporting collaborative learning among students to ensure the progress of learning [25,26,27].

Against this background, what seems clear is that information and communication technologies (ICT) have played an indispensable role in the development of teaching/learning processes at this time and more acutely in these months of pandemic, making possible the deployment of virtual classrooms and classes and generating resilience, creativity, and innovation capacities in teachers during their teaching practice.

However, despite this circumstance, the digital divide has been much more accentuated by the confinement situation [28], which has negatively affected the most disadvantaged young people and children due to the difficulty they have in accessing digital technologies [29]. This situation has highlighted the need to diagnose the socioeconomic and family situations of students to prevent possible existing social inequalities [30]. The role of families behind this problem is relevant since many of them help and support their children’s learning, although it must be taken into account that social, economic, educational, and cultural factors directly influence these circumstances [31], as well as the age of the students or special educational needs [32,33].

With all the above, it is crucial to facilitate learning opportunities and support for teachers, promoting ICT competence and focusing on the design of activities that help students in the acquisition of knowledge through technologies [34,35]. Likewise, it is necessary for educational institutions to respond quickly and always adapted to the context, guaranteeing the continuity and quality of learning processes [36], where the need to support students by creating learning environments has been highlighted [37], meeting their needs and promoting the integration of information technology and education.

Finally, it is necessary to support the well-being of students and their families by providing clear communication about circumstances and expectations, contextualizing each situation, and trying to deal with stressful and confusing situations [38].

## 2. Materials and Methods

The research carried out is qualitative. It is raised from an exploratory, descriptive, and inductive research perspective, close to the data and not generalizable [39], although it may generate future hypotheses [40].

In order to make an adequate approximation to the reality studied, in accordance with the postulates of Jansen [41], a content analysis of the speeches issued by the participants was carried out through the application of the open qualitative survey technique. This research technique is a scientifically based systematic method of collecting information [42], which allows the identification of relevant categories for analysis and facilitates the management and interpretation of the data.

### 2.1. Objective and Research Questions

The main objective was to know the opinion of the directors of different educational centers of the capital of Seville (Spain) about the management carried out in their centers and the possible difficulties encountered during the suspension of face-to-face classes caused by the first wave of the COVID-19 pandemic, which began in March 2020.

As a starting point for the study, the following research questions were posed:

How has the situation caused by COVID-19 affected the organization and management of educational centers?

How has the academic task been developed during the period of cessation of classroom activities?

How has the relationship been with the families of the students during the period of cessation of classroom activities?

### 2.2. Participants

The fieldwork took place from April to June 2020 in the middle of the confinement period in Spain. The selection of participants corresponded to the population of professionals who work as directors of educational centers in Seville (Spain). In the first phase, to achieve the maximum representativeness of the data [43], an intentional sampling was applied under criteria of age, experience in managerial positions, ownership of the center in which they work, teachings that are taught, and the socioeconomic context where the educational center is located. Finally, 43 participants were recruited trying to respect a certain heterogeneity in order to cover all the significant varieties that exist of the phenomenon under investigation.

The sample for this study is made up of 21 men (48.8%) and 22 women (51.2%), with an average age of 50 years and an average experience in managerial positions of 10 years. Fourty-one point nine percent of the participants direct public centers, 32.6% concerted, and 25.6% private. In addition, according to the testimony of the respondents, 69.8% of the educational centers are located in environments considered by the respondents to be of medium socioeconomic level, 25.6% are considered, by the different directors analyzed, of low level, and 4.7% high.

Among the teachings given in these centers, we found the following: Early Childhood Education (18.6%), Primary Education (41.8%), Secondary Education (32.5%), Middle Grade Training Cycles (27.9%), Higher Grade Training Cycles (34.8%), Baccalaureate (23.2%), Adult Education (18.6%), and Special Education (4.64%).

### 2.3. Instrument and Methodological Procedures

For the collection of information, a self-administered questionnaire was used, where open questions of a qualitative nature were included, previously designed, and applied in digital format, facilitating asynchronous data collection [44]. Together with the questionnaire, the participants were provided with all the information related to the purpose of the research, and confidentiality and anonymity were guaranteed, respecting the ethical criteria of qualitative research [45,46].

The methodology carried out in this research, through a rapid distribution, made it possible to obtain responses from the participants on the subject to be studied, being relevant for the present study. Likewise, it is an adequate model since it allowed reducing costs, offering the participants freedom to express their opinions [47,48,49].

In the first section of questions, sociodemographic data were collected (sex, age, experience, ownership of the center in which they work, subjects that are taught, and the socioeconomic context where the educational center is located) that were considered appropriate for the purposes of the research and that allowed us to characterize the sample. Likewise, various questions were posed, divided into three blocks or study dimensions, which sought to evoke ideas that would finally incite the narration of events, lived realities, and personal beliefs about the management of their centers and the possible difficulties encountered during the suspension of face-to-face classes, caused by COVID-19.

The participants responded by writing their opinions and experiences, speeches that were subsequently analyzed with a qualitative research approach. For this, a content analysis of these discourses was carried out, with the intention of discovering the basic components of the studied phenomenon, extracting relevant and convenient information according to the established research objective.

The research instrument (Table 1) was reviewed by different collaborators of the research process according to the intersubjective verification criterion [50] and was designed and validated, in the middle of a pandemic, through the judgment of experts [51,52].

To do this, initially, some criteria for the selection of experts were established based on: (a) academic training related to the subject of study (teachers and pedagogues), and (b) teaching and research experience related to the object of study. Finally, 5 experts were selected.

Next, a rubric for the evaluation of the research instrument was designed to indicate the structural aspects of reliability and validity of the instrument, as well as the content modifications that they considered pertinent. Specifically, we wanted to know the opinion of the expert on the adequacy, clarity, and consistency of the questions, as well as the relevance of the dimensions analyzed. After a first review by the experts consulted, slight modifications were indicated that were applied before the distribution of the instrument to the selected sample. In general terms, the information collection instrument was assessed as suitable, being considered, in the opinion of the experts consulted, as valid for the population to which the research was directed.

The data were systematically processed, according to Jansen [41], through a process of coding differentiated information units at a descriptive level and an axial level, which allowed the identification of a system of codes and sub-codes that correspond to the analysis objectives of the study. After validation by means of adjustments, integration, creation, and restructuring of categories, a system was designed composed of five large codes, which referred to the main groups involved in the analysis, and eleven sub-codes, which referred to the features, dimensions, or elements identified in the speech (Table 2).

### 2.4. Analysis and Treatment of Information

To analyze the information obtained, the qualitative data analysis software ATLAS.ti 9 was used. At the beginning of the process, the first set of codes was generated from the open coding of information segments or units in an inductive manner [53], which were subsequently refined with successive coding establishing relationships between analysis codes.

### 2.5. Concordance between Coders in Research

The reliability of the set of categories was calculated through the analysis of agreement between coders to ensure the consistency of the data and the accuracy of the investigation. To carry out the measurement, 4 independent coders participated, directly related to the subject of study and external to the research. Once the coders provided the relevant information, the Fleiss Kappa index was used to find the agreement coefficients, yielding a value of k = 0.712 that represents a good agreement strength [54]. On the basis of this data, it can be stated that there was a high agreement between coders as there was agreement in the recognition of the codes of the same information units provided.

## 3. Results and Discussion

The people surveyed provided a large number of opinions and perspectives about the management of educational centers in the midst of the COVID-19 pandemic, identifying negative aspects, facilitating positive aspects, and reflecting on the socio-educational implications of this pandemic, in an important volume of information that needs to be uncovered. In this regard, first, we present and analyze the tables of re-count of citations and percentages of the codes and subcodes (Table 3 and Table 4).

Based on the informants’ speeches, it can be affirmed that there was repeated reference to the negative aspects that arose in the educational centers during the period of cessation of classroom activities (few guidelines by the educational Administration, scarce technological resources available to students, etc.). Similarly, considering the count of citations, it was observed how the informants frequently alluded to the students in their speeches, emphasizing questions related to the way of responding to the new situation of online teaching–learning of the students, or the possible adaptations that were carried out with certain students, among other matters.

On the other hand, according to the data provided by the interviewees, the allusions related to families, the educational Administration, and technological resources had an estimable count of citations.

Second, and continuing with the analysis, the co-occurrence between codes is presented and examined (Table 5).

Considering the content provided by the informants, relationships were established between codes that facilitate the interpretation of the results. Observing Table 5, we can verify that, when the informants mentioned the educational Administration, they related it mainly to the negative aspects (*n* = 19), with information (*n* = 9), and with technological resources (*n* = 8).

One can state that the directors, and directors surveyed, focused in their speeches, especially on the difficulties suffered by the centers in really knowing the exact situation, interacting with the academic authorities, and attending, even from a distance and using ICT, to the students.

Indeed, if we look at the “Students” code, a fairly high association (*n* = 20) stood out with the negative aspects that arose during the cessation of classroom activities, also highlighting a clear relationship with the codes “Technological resources” (*n* = 12) and “Positive aspects” (*n* = 10).

On the other hand, the code “Families” had a high co-occurrence with the codes “Negative aspects” (*n* = 18) and “Technological resources” (*n* = 13).

In the visualization and analysis of codes that met the condition of concurring with other codes, we underline as highly significant those related to the negative aspects and the positive aspects highlighted by the informants in relation to their opinions about the situation experienced by the centers and the community education in times of pandemic.

**Figure 1:**
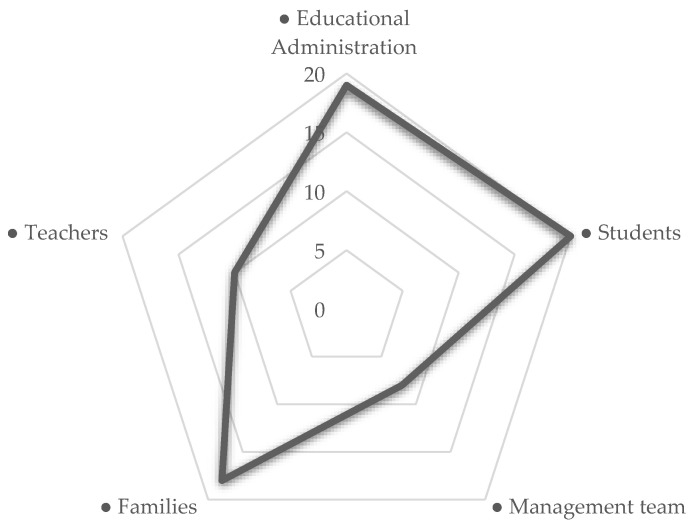
Co-occurrences between the “Negative aspects” code and the rest of the codes.

Thus, when the informants alluded to the negative aspects that occurred during the period of cessation of classroom activities (Figure 1), they related these in the first place to the educational Administration and the students. In the same way, the negative aspects were also linked, always in the opinion of the respondents, with the families, and, to a lesser extent, with the teaching staff.

It is powerfully striking how the directors participating in these speeches did not relate the negative aspects detected with their work as managers of centers and human teams (“Management team” code).

**Figure 2:**
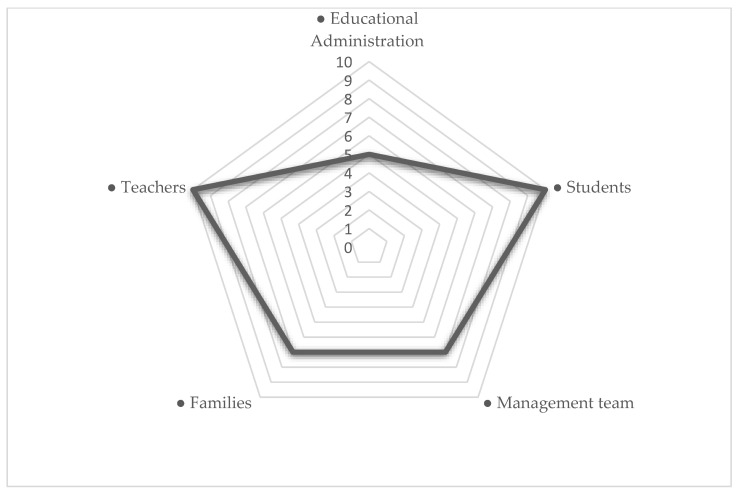
Co-occurrences between the “Positive aspects” code and the rest of the codes.

If the co-occurrences between the “Positive aspects” code and the rest of the codes are studied (Figure 2), it can be affirmed that these were clearly related to students and teachers, highlighting the step forward and sacrifice, commitment, and involvement of these two sectors of the educational community involved in this extreme situation. In the same way, when the informants alluded to the positive questions generated during the period of cessation of classroom activities, they related it with the same frequency with the management team and the families, but to a much lower degree.

In addition, observing the co-occurrence in relation to the negative and positive aspects manifested by the main people involved in the analysis, it can be seen how the major negative issues that occurred were directly related to the educational Administration (*n* = 19), followed by aspects related to the teaching staff (*n* = 10), with the families (*n* = 6) and, to a lesser extent, with the students (*n* = 5), and with the management team (*n* = 4).

If we pay attention to the positive aspects contemplated by the respondents, it can be intuited that they were related to the same extent with the codes “Educational Administration” (*n* = 5) and “Management team” (*n* = 7). On the other hand, the code “Students” (*n* = 10) and “Families” (*n* = 7) had a direct relationship with these positive aspects, as well as the code “Teachers” (*n*= 10).

**Figure 3:**
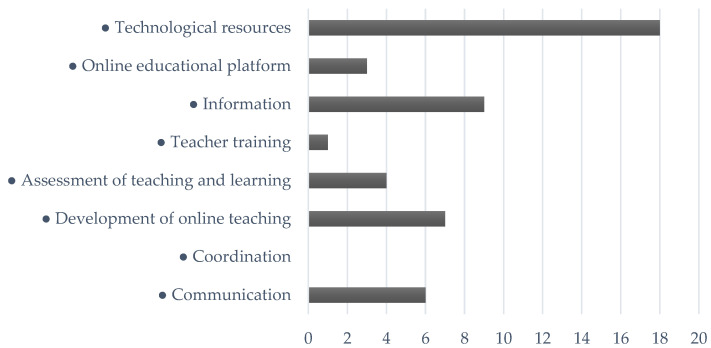
Co-occurrences between the “Positive aspects” code and the rest of the codes.

Next (Figure 3), the co-occurrences between the code “Negative aspects” and the different subcodes analyzed were observed. It can be stated that the main negative issues that occurred were directly related to technological resources. Specifically, the informants alluded to difficulties already mentioned derived from the scarce training for its use, as well as the limited technological infrastructures that educational centers and families may have at home.

In another vein, the negative aspects described by the research participants were related to the information provided by the educational Administration. Specifically, they alluded to the fact that it had been scarce and late, as we have pointed out in previous pages.

Another of the problems especially manifested was directly related to the development of online teaching. The principals surveyed indicated that the transition from classroom to online teaching had been an arduous task where they had to face multiple impediments.

Likewise, communication was a negative aspect to highlight in this section of subcodes since the respondents unequivocally declared that the cessation of classroom activities had hindered communication between the various educational agents, being an obstacle to the optimal development of the process of teaching–learning.

Continuing with the analysis, the respondents pointed out the evaluation of teaching and learning as a negative aspect since, as indicated previously, due to the haste of the measures to be adopted, the evaluation criteria had to be urgently reformulated.

Finally, it is necessary to highlight that teacher training was not considered by those surveyed as a negative aspect, despite its importance as an essential element for educational success.

Third and last, the analysis of the results regarding the content of the informants’ speeches is presented in relation to the situation experienced in educational centers by teachers, students, and families throughout this period of pandemic, highlighted especially by the directors surveyed.

### 3.1. Teachers

The respondents, when referring to the situation experienced by the teachers of their respective centers in this time of pandemic, clearly expressed problems related to the development of online teaching that they had to face urgently and hastily, especially with the form and evaluation criteria of its students.

“The situation has been (and continues to be) quite complicated due to the uncertainty that teachers and students are experiencing, without knowing when and how it was going to be evaluated (in my case ESA students).”

(Informant 15, Woman, 3 years of experience, Public Center, Socioeconomic level of the Low context)

“Being Professional Training, the practical part cannot be taught by correcting the students in the learning process nor can it be evaluated objectively.”

(Informant 18, Woman, 1 year of experience, Concerted Center, Socioeconomic level of the Medium context)

Similarly, we also found in the various speeches of the respondents very varied information that indicated the difficulty of communicating with their students in an adequate way, obtaining feedback during the development of non-face-to-face teaching, generating trust and group cohesion, fostering relationships, interpersonal skills, and the handling of all kinds of conflicts that arose during the process of adapting to a teaching format so different from the one that had been experienced in person in the classrooms of educational centers, sometimes in a very precarious and unvariable way.

“Nothing can replace the conditions that occur in face-to-face teaching, the relationship of closeness, safety and comfort (the blackboard is a fundamental instrument) that occurs in the classroom. Online teaching has many disadvantages.”

(Informant 15, Woman, 3 years of experience, Public Center, Socioeconomic level of Low context)

Another negative aspect, which also appeared repeatedly in the various statements, was related to the sometimes deficient training in technologies for education and the access and availability of material resources to develop them. Similarly, it became clear how the teachers themselves and the students’ families did not always have these technological resources available, seriously damaging their situation.

“(An important problem detected is…) The training of teachers, students and families in distance education platforms.”

(Informant 17, Male, 7 years of experience, Public Center, Socioeconomic level of the Low context)

“Teachers are asked to use the internet (teachers must have internet at home and this is not necessarily the case, for example, teachers for rent may only have mobile internet with mega restrictions).”

(Informant 2, Female, 2 years of experience, Concerted Center, Socioeconomic level of the Medium context)

Despite all these deficiencies, the teachers solved the difficulties by relying on their passion and teaching vocation, on their creativity and ability to adapt to difficulties, creating spaces for mutual learning, relying on coordinated interdisciplinary work teams, and pursuing at all times a fluid and empathetic communication between teachers, as well as with families and students.

“The vocation and work of the teaching staff is what has made all this work from the first hour.”

(Informant 10, Woman, 2 years of experience, Public Center, Socioeconomic level of the Middle context)

“The entire faculty has reacted quickly and effectively by creating virtual spaces for coordination of teaching teams and communications with families and students..”

(Informant 14, Woman, 2 years of experience, Public Center, Socioeconomic level of the High context)

### 3.2. Students

Regarding the problems that directly affected students, at different levels of education, the surveyed people warned that there was a significant number of students who did not have sufficient technological resources to be able to carry out adequate monitoring of the training process, pointing out the deficiencies of a significant percentage of students in relation to internet access, the impossibility of having the necessary technological resources to follow an online teaching–learning process, the conditions of family and home overcrowding of some students, as well as the circumstance that many students had to share resources and devices with parents and siblings, greatly hindering their educational development.

“I am especially concerned about those students who do not have the internet at home, 10–15% of our total student body, being impossible for them to monitor the teaching activity.”

(Informant 5, Woman, 5 years of experience, Public Center, Socioeconomic level of the Low context)

Especially affected are students with special educational needs, where the consequences of such an abrupt change in the way of imparting and receiving teaching greatly impaired their development and the attention to diversity they require.

“Students with learning difficulties are the ones we consider to be having the greatest negative impact.”

(Informant 19, Woman, 10 years of experience, Concerted Center, Socioeconomic level of the Medium context)

On the other hand, the respondents indicated that the development of practical educational activities, which were part of the formal curriculum of some centers, had been seriously affected by not being able to adapt correctly to an online format, caused by abrupt and immediate confinement.

“It is very difficult for a student to do their internship at home.”

(Informant 1, Male, 30 years of experience, Private Center, Socioeconomic level of the Medium context)

In the same way, the participants in the research expressed that the students had shown an enormous capacity to adapt to non-face-to-face teaching, assuming that it had been a situation of high health severity that required all people to be involved in maintaining certain adequate security measures to curb the impact of the virus on the population.

The relational and social aspect between students and teachers was key, collaborating with each other to progress in the training processes, trying to reduce the collateral damage of an urgently enabled teaching methodology.

“Both students and teachers have shown a capacity to adapt to admirable circumstances.”

(Informant 3, Woman, 9 years of experience, Private Center, Socioeconomic level of the Medium context)

“As for the teachers and students, they have adapted to this new way of teaching in a surprising way, collaborating with each other to make it as easy as possible, with full attendance of all classes. Despite the difficulty that this type of teaching entails, we believe that this has served in part to make the students become more involved in their teaching and collaborate, and have a better relationship with the teachers.”

(Informant 20, Woman, 10 years of experience, Private Center, Socioeconomic level of the Medium context)

### 3.3. Families 

If we look at the informants’ discourse on the negative aspects that directly affected the families of the students, we observe that, once again, access to technological resources for each family was key in the educational response to the pandemic, as well as the lack of training by parents in digital skills, especially in educational settings.

This circumstance entailed a reflection on the part of the informants on the diverse socioeconomic and cultural possibilities of the families, which generated educational difficulties for the students and undermined the possibilities of access in equal conditions, increasing an unequal social failure that alienated the students with fewer resources and caused a clear differentiation between centers.

“Students do not have to have devices at home to be able to connect, or they may have a device, but they have to share it with siblings or parents who have to telework.”

(Informant 2, Woman, 2 years of experience, Concerted Center, Level socioeconomic of the Middle context)

“What has been called the ”digital divide” the main problem asserts itself in terms of scarcity of technological resources (…) and the lack of preparation of the vast majority of mothers and fathers for the proper use of ICT in teaching–learning situations.”

(Informant 24, Male, 13 years of experience, Public Center, Low socioeconomic level)

As we advanced previously, another important aspect is the level of digital competence that families have, as well as their academic training and cultural level. The heterogeneity of situations in this sense is something to take into account, since not all parents were prepared to support their children in academic tasks, together with the fact that many of them had to telework and share spaces and resources, on occasions crowded, for months, in their homes at the time of confinement.

“Not all students or families have to have a sufficient level of digital competence to access the materials used.”

(Informant 2, Woman, 2 years of experience, Concerted Center, Socioeconomic level of the Middle context)

“(Another problem detected has been…) ICT literacy of families and students.”

(Informant 4, Male, 8 years of experience, Public Center, Low socioeconomic level of the context)

“The unequal access to tasks is very important due to various circumstances (family, resources, etc.) and the difficulty in an evaluation.”

(Informant 12, Woman, 5 years of experience, Concerted Center, Socioeconomic level of the Middle context)

In addition, there are families not particularly involved in the education of their children under normal conditions and, even less, in a situation as extraordinary as that caused by the pandemic, which has favored a greater delay in their children’s learning.

“Likewise, with family involvement (they allow their children to get up late, they are not aware if their children perform the required tasks, etc.).”

(Informant 2, Woman, 2 years of experience, Concerted Center, Socioeconomic level of the Middle context)

Despite the difficulties, the educational centers have provided the families of their students, as far as possible, with access to technological resources or alternative ways to develop classes.

“The problem has arisen with families who had difficulty with technological means, but they have been solved, in most cases.”

(Informant 19, Woman, 10 years of experience, Concerted Center, Socioeconomic level of the Middle context)

## 4. Conclusions

Thanks to the analysis carried out, various conclusions can be drawn about the situation that teachers, students, and families experienced in this time of pandemic.

The abrupt closure of schools [9,11] made necessary a rapid conversion from face-to-face education to distance education [12], without time to plan a strategy and the worsening of the digital divide between some students and others. This study offers the possibility of understanding a concrete reality contextualized in a time of educational transition in the face of the uncertainty of a pandemic that has affected the world population in multiple aspects.

The uncertainty experienced by teachers, students, and families in this time of pandemic is highlighted, as well as the difficulty to establish fluid communication with students in this new educational scenario, the need for distance education training, and overcoming the problems of access to technological resources. In this sense, the opinions analyzed corroborate what was stated by other reference authors [20,21] when they affirm that many students do not have the technological resources necessary to carry out an online learning process. In the study carried out, this situation of lack of access to technology is especially highlighted with the students of publicly-owned centers located in environments with a low socioeconomic level. Respondents mentioned some factors that hindered online educational development, such as inaccessibility to the internet or the fact that some students had shared technological devices with other family members.

In the same way, the directors alluded to the difficulties of students with special educational needs, to follow their educational development in an optimal way and considering the particularities that they present.

On the other hand, it is highlighted that, on some occasions, the use of ICT had not guaranteed that communication with students was fluent, which prevented the establishment of fruitful interpersonal relationships and individualized monitoring of students’ needs. In this sense, permanent interaction between teachers and students is extremely important, and interaction that guarantees adequate feedback, generating trust in the teaching–learning process and favoring group cohesion [14,24].

Another of the aspects mentioned in the study deals with training and access to information and communication technologies by teachers [35]. Specifically, the informants affirmed that, on occasions, this ICT training was not enough, hindering the optimal development of teaching, as well as their, on occasions, poor access to appropriate technological resources, aspects that clearly need to be improved in the future. 

The results obtained show that despite the obstacles encountered by the suspension of face-to-face classes during the first wave of the COVID-19 pandemic, those involved in the process expressed a positive attitude, highlighting the adaptability of humans to the challenges that arise on a day-to-day basis.

Regarding the information provided by the respondents on families, the data indicated that, once again, access to technological resources had been a determining factor in the educational process. In this sense, the lack of digital skills on the part of parents has once again generated inequalities among students [31]. The analysis carried out indicated that families with more technological and cultural resources have been able to better support their sons and daughters in educational tasks.

We understand that the data in this article cannot be generalized due to the size of the sample. Therefore, as a future line of research, it is intended to increase the sample with the opinions of other directors of educational centers from different territories of Spain, which would allow us to understand how the situation caused by COVID-19 has affected the organization and management of educational centers. Likewise, we understand that we could go deeper into aspects, such as the organization and management of educational centers during the period of “new normality”, examining how the academic task has been developing in this new context.

In summary, in the face of this new uncertain socio-educational scenario, it is essential to reflect on the defining characteristics of the teaching profession and its practices, the importance of digital training, collaboration with families, and the guarantee of minimum technological resources that ensure access to education on equal terms. 

## Figures and Tables

**Table 1:** Blocks and questions of the questionnaire.

Blocks	Questions
I. Organization and management from the management team during the period of cessation of classroom activities	How is the coordination of the management team during the period of cessation of classroom activities?
	Does the educational center have the necessary technological resources to be able to respond to the problems that arise during the cessation of classroom activities?
	Are the information and resources provided by the Ministry of Education, Culture and Sports of the Junta de Andalucía adequate?
	Do the teachers who are part of the educational teams of the center have the necessary digital training to face this situation?
II. Development of the academic task during the period of cessation of classroom activities	Does the center use any online educational platform for academic development during this situation? If so, which one/is it?
	Have the teachers had difficulties teaching their classes online?
	How do students respond to the new online teaching–learning situation?
	Have students with special educational needs been affected by the situation?
	Has the evaluation of the students’ learning processes developed normally?
III. Relationship with families during the period of cessation of classroom activities	Is communication with the students’ families adequate?
	Do families have the necessary technological resources to face the situation?
	Have families expressed concern about having to support their sons and daughters in academic activities during this period of time?

Source: self-made.

**Table 2:** Codes and subcodes derived from data analysis.

Codes	Subcodes	Subcodes
	(Level 1)	(Level 2)
Teachers	Negative aspects	Communication
		Information
Families	Neutral aspects	Development of online teaching
Management team		Online educational platform
Students	Positive aspects	Teacher training
Educational Administration		Technological resources
		Coordination
		Assessment of teaching and learning

Source: self-made.

**Table 3:** Re-count of citations and percentages of codes and subcodes level 1.

		n	% Respect to the Total of Appointments of Each Encoding Level
**Codes**	Teachers	22	19.64%
	Families	23	20.53%
	Management team	15	13.39%
	Students	29	25.89%
	Educational Administration	23	20.53%
	Total	112	100%
**Subcodes** **(Level 1)**	Negative aspects	41	36.6%
	Neutral aspects	52	46.42%
	Positive aspects	19	16.96%
	Total	112	100%

**Table 4:** Re-count of citations and percentages of subcodes level 2.

		n	% Respect to the Total of Appointments of Each Encoding Level
**Subcodes** **(Level 2)**	Communication	9	8.03%
	Information	9	8.03%
	Development of online teaching	10	8.92%
	Online educational platform	3	2.67%
	Teacher training	3	2.67%
	Technological resources	23	20.53%
	Coordination	3	2.67%
	Assessment of teaching and learning	5	4.46%
	Others not related to the research objectives	47	41.96%
	Total	112	100%

**Table 5:** Co-occurrence count between codes.

		Subcodes		Subcodes *							
		(Level 1)		(Level 2)							
		Negative Aspects	Positive Aspects	COM	COO	DOT	ATL	TT	INFO	OEP	TR
Codes	Educational Administration	19	5	5	1	3	1	1	9	0	8
	Students	20	10	4	2	9	4	3	2	2	12
	Management team	8	7	3	2	1	0	1	3	0	5
	Families	18	7	5	2	3	1	2	3	1	13
	Teachers	10	10	4	3	6	4	3	0	2	7

* Note: Communication: COM/Coordination: COO/Development of online teaching: DOT/Assessment of teaching and learning: ATL/Teacher training: TT/Information: INFO/Online educational platform: OEP/Technological Resources: TR.
